# Public healthcare practitioners’ knowledge, attitudes and practices related to oral antibiotic prescriptions for dental use in Pietermaritzburg, KwaZulu-Natal

**DOI:** 10.4102/hsag.v27i0.1832

**Published:** 2022-04-29

**Authors:** Prishana Ramnarain, Shenuka Singh

**Affiliations:** 1 Discipline of Dentistry, School of Health Sciences, University of KwaZulu-Natal, Durban, South Africa

**Keywords:** dental conditions, health practitioners, oral antibiotics, prescriptions, public health

## Abstract

**Background:**

There is limited published evidence on health workers’ perspectives on trends in oral antibiotic prescription for dental conditions in the public health sector.

**Aim:**

This study set to determine healthcare practitioners’ knowledge, attitudes and practices related to oral antibiotic prescriptions for dental use.

**Setting:**

This included two public hospitals in Pietermaritzburg.

**Methods:**

This was a cross-sectional study using quantitative data. Purposive sampling was used to select medical and dental practitioners from Institution A and B (*n* = 122). A self-administered questionnaire was developed using open and close-ended questions. Data were collected and analysed using the Statistical Package for Social Sciences (IBM SPSS version 25R).

**Results:**

The response rate for the study was 72.1%. The majority of study participants (*n* = 72, 81.8%) indicated awareness of an antibiotic stewardship programme in their respective institutions. However, a significant number (*n* = 42; 47.7%) of participants were unsure of whether this programme was active. Most participants (*n* = 80, 90.9%) indicated the need for improving oral antibiotic prescription for dental conditions. Participants indicated prescription of antibiotics for orofacial swellings (*n* = 52; 59.0%) and dental pain related to irreversible pulpitis (*n* = 29; 32.9%), reversible pulpitis (*n* = 33; 37.5%) and dental fillings (*n* = 15; 17.0%). Antibiotics were also prescribed for pericoronitis (*n* = 58; 65.9%), periodontitis (*n* = 57; 64.7%) and impacted teeth (*n* = 21; 23.8%). All dental practitioners (*n* = 14) supported the need for antibiotic cover for pericoronitis and periodontitis.

**Conclusion:**

The results indicated inconsistencies in healthcare practitioners’ reported knowledge, attitudes and practices related to antibiotic prescription patterns.

**Contribution:**

This study highlights the need for clear evidence-based guidelines for antibiotic prescription for dental conditions.

## Introduction

Antibiotics have played a major role in the management of infectious diseases; however, limited access in the availability of affordable antibiotics, especially in developing countries, remains a challenge (Watkins et al. 2019). At the same time, there is a reported increase in the use of antibiotics for dental clinical management (Haliti et al. 2017). From a South African dental management perspective, antibiotics appear to be most commonly prescribed for orofacial and dental infections (Huang & Owen 2012). The overuse and misuse of antibiotics for health conditions, including dental conditions that do not require such interventions, place further strain on the already scarce resources, especially in the public health sector (Mthethwa & Matjila 2018). Studies further show that the overuse of antibiotics has collectively contributed to the emergence of resistant bacterial strains, and that this has necessitated a renewed focus on a post-antibiotic era (Boyles et al. 2017; Hoffman et al. 2015; Mendelson & Matsoso 2015). This post-antibiotic era highlights the need for managing the emergence of antimicrobial resistance through monitoring and a review of current trends and patterns in antibiotic prescriptions (Carter, Sun & Jump 2016; Crowther-Gibson et al. 2011; Truter 2015). A well-coordinated antibiotic stewardship programme could be one of the possible public health strategies to ease the burden on the public healthcare system. Likewise, dental health workers in South Africa need to be aware of the impact of antimicrobial resistance and how this could affect clinical management of dental conditions, as well as clinical outcomes (Mthethwa & Matjila 2018).

### Trends in antibiotic prescriptions for dental use

Not much published evidence exists in South Africa on the related trends and patterns in antibiotic prescriptions for dental use; however, studies show that there are inconsistencies in how dental conditions are managed, specifically in relation to the indications for antibiotic use. The following studies highlight some of these inconsistent trends in antibiotic prescriptions. Sancho-Puchades et al. (2009) observed a mismatch between the practice of prescribing antibiotic prophylaxis for the prevention of local odontogenic infections and the recommended associated guidelines for dental clinical management. Similarly, other authors pointed out that overprescription of antibiotics occurred even when service providers such as dentists had adequate knowledge on the indications and contraindications of antibiotic use (Carter et al. 2016; Truter 2015). At the same time, Swiss dentists reported not being sure of when to prescribe antibiotics (Sweeney et al. 2004). This observation is not unique. While it might appear that dental practitioners are prescribing antibiotics judiciously in South Africa, according to Lalloo et al., there was no consensus amongst these practitioners on when and why to prescribe antibiotics for dental use (Lalloo et al. 2017a). The implications of these findings are that there could be inconsistencies in antibiotic prescription trends for dental use (Ncube et al. 2017).

This study arose owing to the limited available data on the trends and practitioners’ perceptions and attitudes towards antibiotic prescription in South Africa, specifically in KwaZulu-Natal (Ramnarain 2021). There are limited available data on the patterns of antibiotic prescription for dental conditions and of the treatment outcomes in terms of statistical records in the public oral health sector. This creates an unclear picture of prescription trends and patterns on oral antibiotics for dental conditions in KwaZulu-Natal (Ramnarain 2021). The resultant impact of the current trends in dental management in relation to antibiotic prescriptions and the possible antimicrobial resistance are largely unknown (Ramnarain 2021). To this end, the aim of this study was to determine healthcare practitioners’ knowledge, attitudes and practices related to antibiotic prescription for dental purposes in the Pietermaritzburg region of KwaZulu-Natal.

### The Pietermaritzburg complex

Pietermaritzburg, which is located in the uMgungundlovu district, is considered the second largest city in KwaZulu-Natal with a population of about 11 527 96 people (Statistics South Africa 2013–2018). In addition, about 925 695 people in the uMgungundlovu district remain uninsured (Statistics South Africa 2013–2018), thus creating a huge burden on the public health system for health service provision (KwaZulu-Natal Department of Health 2015). Similarly, there is a huge dependency on the public oral health system to provide oral healthcare to the majority of the population; yet, only a small fraction of the oral health workforce is located in this sector (Singh 2011; Singh, Myburgh & Lalloo 2010; Thema & Singh 2017).

From a service delivery perspective, public oral healthcare can be accessed at the district, regional or provincial tertiary levels. The district- and regional-level facilities offer services for the management of oral conditions such as dental caries, dental impactions, periodontal disease, trauma and various types of oral pathology, while provincial tertiary-level hospital offers specialised maxillofacial services (KwaZulu-Natal Department of Health 2018). These include the management of maxillofacial injuries caused by motor vehicle accidents, assaults, sports injuries and gunshot wounds. Pathology related to the maxillofacial area is also managed at this level (KwaZulu-Natal Department of Health 2018).

## Methods

This was a cross-sectional study using quantitative data to determine healthcare practitioners’ knowledge, attitudes and practices related to oral antibiotic prescriptions for dental use (Ramnarain 2021). The research site was the Pietermaritzburg Complex in KwaZulu-Natal. Two public oral health facilities were purposively selected for the study (Institutions A and B).

Purposive sampling was also used to select the study sample (*n* = 122). The sample comprised 108 healthcare workers and 14 oral healthcare workers (Table 1). The selection criteria included all healthcare professionals who prescribed and dispensed antibiotics for dental use. The study excluded all dental and medical managers and practitioners not involved in the clinical management and prescription of antibiotics for dental use (Ramnarain 2021). A written consent was first obtained from all participants, and ethical issues such as confidentiality and anonymity were upheld.

**TABLE 1:** Study participants (*N* = 22).

Staff	No. of participants
**Medical**	
Surgeons	6
Medical officers	37
Emergency medical specialists	2
Emergency medical officers	8
Interns	39
Specialists	4
Registrars	4
Clinical managers	4
Anaesthesiologists	4
Total	108
**Dental**	
Dentists	8
Dental therapists	6
Total	14

A self-administered questionnaire with open- and closed-ended questions was developed for the study. The questionnaire comprised 15 questions that were designed to assess health workers’ knowledge, perceptions, attitudes and practices related to antibiotic prescriptions for dental use. The first part of the questionnaire included socio-demographic data, such as gender and employment. The questionnaire focused on participants’ knowledge of guidelines developed by National Institute for Health care and Excellence and the guideline by the American Heart Association, the South African National Strategic Framework on Antimicrobial Resistance and antibiotic stewardship (Ramnarain 2021).

The next part of the questionnaire focused on the health practitioners’ practices related to antibiotic prescriptions for dental use. Participants were asked to indicate if they would prescribe oral antibiotics for specific dental conditions such as orofacial swellings, pulpitis and mandibular fractures or maxillary fractures.

The last part of the questionnaire used open-ended questions to explore practitioners’ perceptions and attitudes towards the current trends in antibiotic prescription. Participants were also asked to make recommendations on how to improve the trends in antibiotic prescription for dental use (Ramnarain 2021).

Data were first cleaned and coded. A code book was prepared to ensure that codes were exclusive. Participants were also, provided code names such as P1, P2, etc. Data were then captured onto an Excel spread sheet and analysed using the Statistical Package for Social Sciences’ (IBM SPSS Version 25^R^). Univariate descriptive statistics, such as frequency and mean distribution, were conducted for all variables. Bivariate statistics was also be used to assess the outcome, and the outcome was analysed by the explanatory variable (Bertani et al. 2018). Inferential techniques included Pearson’s chi-square test to assess a possible relationship between the independent variable (employment or occupation) and dependent variable (antibiotic prescription). A *p*-value of 0.05 was established as being statistically significant.

Thematic analysis was used for the open-ended questions. The responses were first read by the researcher several times to gain familiarity with the data (making connections in the data). For the subsequent steps, the data were coded and collated so that emergent themes could be further examined and compared for possible associations (through reflection, critical thinking and understanding).

### Ethical considerations

Ethical clearance was obtained from the University of KwaZulu-Natal (BREC Reference number: BE026/19). Gatekeeper permission and approval were obtained from the KwaZulu-Natal Department of Health (Reference No.: NHRD Ref: KZ_201902_018).

## Results

Only 88 participants returned the completed questionnaire, yielding a response rate of 72.1%. The majority of study participants were female (*n* = 59; 67.0%) and employed as medical practitioners (*n* = 74; 84.1%), while only 2.3% were community service dentists (*n* = 2).

### Knowledge of oral antibiotic prescriptions

An overwhelming majority of participants (*n* = 85; 96.5%) were aware of the Standard Treatment Guidelines and the Essential Medicines List. Similarly, a large number of participants (*n* = 73; 82.9%) were aware of the American Heart Association guidelines, while more than half of the study sample (*n* = 53; 60.2%) was aware of the National Institute of Health Care and Excellence guidelines.

More than two-thirds of the study sample (*n* = 72, 81.8%) indicated awareness of an antibiotic stewardship programme in their respective institutions. However, a significant number of participants (*n* = 42; 47.7%) were unsure of whether the institutional programme was active.

### Practitioner perceptions and attitudes towards oral antibiotic prescriptions

The majority of participants (*n* = 80, 90.9%) indicated that there was a need for improving oral antibiotic prescription for dental conditions.

Some of the emergent themes that arose from analysis of the open-ended questions included inappropriate prescription of antibiotics and limited availability of appropriate antibiotics. Respondents pointed out that overprescription of antibiotics occurred or that antibiotics were prescribed for conditions not requiring such intervention as reflected by the following quotes:

‘Antibiotics are sometimes prescribed for viral illness.’ (P108, female, aged 33, dentist)

‘Over-prescription of antibiotics with insufficient diagnosis.’ (P62, male, aged 52, medical practitioner)

The qualitative data analysis further indicated that antibiotics are prescribed mainly for prophylaxis purposes. The limited availability of appropriate antibiotics could also influence the practitioner’s pattern of antibiotic prescriptions, as indicated by the following quote:

‘Limited spectrum of medication or antibiotics available after hours when the pharmacy is closed.’ (P23, female, aged 42, medical practitioner)

Other recommendations included proper use of prescription guidelines when prescribing antibiotics and proper monitoring and control of drug dosage.

### Antibiotic prescription practices

More than half of the study sample (*n* = 60; 68.1%) indicated referring to the Standard Treatment guidelines ‘some times’ when prescribing antibiotics. At least half of the study sample (*n* = 44; 50%) followed the American Heart Association guidelines when prescribing antibiotics for the prevention of infective endocarditis, while 26 (29.5%) followed the National Institute for Health Care and Excellence guidelines for the same condition. More than half of the study sample (*n* = 52; 59.0%) indicated that they would prescribe antibiotics for orofacial swellings, while 15 health practitioners (17.0%) were not sure (Table 2).

**TABLE 2:** Dental conditions requiring antibiotics from healthcare practitioners’ perspective.

Dental condition	Study sample response *n* = 88 (%)						Healthcare practitioners			
	Yes		No		Unsure		Medical practitioners (*n* = 74)		Dental practitioners (*n* = 14)	
	*n*	%	*n*	%	*n*	%	Yes	No	Yes	No
Orofacial swelling	52	59.0	21	23.8	15	17.0	38	21	14	0
Dental pain related to irreversible pulpitis	29	32.9	42	47.7	17	19.3	24	33	5	9
Dental pain related to reversible pulpitis	33	37.5	36	40.9	19	21.5	32	23	1	13
Dental pain related to dental fillings	15	17.0	57	64.7	16	18.1	14	44	1	13
Pericoronitis	58	65.9	13	14.7	17	19.3	44	13	14	0
Periodontitis	57	64.7	13	14.7	18	20.4	43	13	14	0
Impacted teeth	21	23.8	48	54.5	19	21.5	13	42	8	6
Acute alveolar osteitis or dry socket	39	44.3	22	25.0	27	30.6	31	20	8	2
Mandibular fractures or maxillary fractures	46	52.2	22	25.0	20	22.7	32	22	14	0
Trismus	31	35.2	39	44.3	18	20.4	23	33	8	6
Prophylaxis before dental surgery	56	63.6	18	20.4	14	15.9	46	14	10	4
Postoperative treatment for dental surgery	41	46.5	26	29.5	21	23.8	34	19	7	7
Aphthous ulcers	16	18.1	50	56.8	22	25.0	15	37	1	13

Likewise, study participants indicated antibiotic cover for dental pain related to irreversible pulpitis (*n* = 29; 32.9%), reversible pulpitis (*n* = 33; 37.5%) and dental fillings (*n* = 15; 17.0%). With regard to the management of dental pain related to irreversible pulpitis, the following results were obtained from an institutional perspective. About 20 participants (57.1%) from Institution A and 16 (30.1%) from Institution B would prescribe antibiotics for this dental condition (*p* = 0.029). Interestingly, the majority of dental practitioners in the study sample indicated that antibiotic cover is not needed for irreversible pulpitis (*n* = 9), reversible pulpitis (*n* = 13) and dental fillings (*n* = 13).

Study participants also indicated prescription of antibiotics for pericoronitis (*n* = 58; 65.9%), periodontitis (*n* = 57; 64.7%) and impacted teeth (*n* = 21; 23.8%). From an institutional perspective, 31 participants (88.9%) from Institution A and 40 (75.0%) from Institution B would prescribe antibiotics for pericoronitis. Similarly, 27 participants (76.9%) from Institution A and 14 (72.1%) from Institution B would prescribe antibiotics for periodontitis. All dental practitioners (*n* = 14) supported the need for antibiotic cover for pericoronitis and periodontitis.

Only 32 health practitioners would prescribe antibiotics for maxillary and mandibular fractures, while all dental practitioners (*n* = 14) indicated the need to do so but 20 study participants were unsure (22.7%). A total of 34 health practitioners indicated the need for antibiotic cover for post-operative dental surgical treatment, while only 50% of dental practitioners (*n* = 7) supported this. It was noteworthy that more than half of the study sample (*n* = 50; 56.8%), including the majority of dental practitioners (*n* = 13) would not prescribe antibiotics for the management of aphthous ulcers.

Less than half of the study participants (*n* = 43, 40%) indicated that sometimes they would review their clients in a follow-up visit after antibiotic prescription.

## Discussion

The study findings suggested general inconsistencies in health practitioners’ knowledge and practices related to antibiotic prescriptions for dental use. Participants in this study were aware of the Standard Treatment guidelines and the Essential Medicines List (96.5%), the American Heart Association guidelines (82.9%) and the National Institute of Health Care and Excellence guidelines (60.2%); yet, differences were observed in the prescription trends for the same dental condition. About 68.1% of the study sample indicated referring to the Standard Treatment guidelines ‘sometimes’ for the prescription of oral antibiotics. In contrast, a previous study reported that only 45% of practitioners adhered to the Standard Treatment guidelines and Essential Medicines List in the Primary Health Care in South Africa (Gasson 2018). The study findings are consistent with Marra et al. who also observed differences in antibiotic prescription trends in their study (Marra et al. 2016). Likewise, Mthethwa and Matjila reported that dentists in their study were aware of the treatment guidelines but few followed the recommendations for antibiotic prophylaxis (Mthethwa & Matjila 2018). According to Gajdács et al. (2020), healthcare practitioners had proper levels of knowledge on antibiotic therapy but were less familiar with the patho-mechanisms of infectious diseases and bacterial resistance. The implication of this finding is that adequate practitioner knowledge does not guarantee proper prescription practices because many reports have also suggested that practices are mainly influenced by the attitudes of the healthcare practitioners.

At the same time, Lalloo et al. (2017b) postulated that antibiotic prescription patterns do not appear to follow a coherent set of guidelines or sound indications for antibiotic use. Inconsistencies in the recognised guidelines for antibiotic prescriptions have added to this confusion (Ramnarain 2021). The National Institute for Health Care and Excellence changed the routine prescription of antibiotics for the prevention of infective endocarditis in 2008; however, the American and European cardiologists still recommend the use of antibiotic prophylaxis for the prevention of same condition (Bunce & Hellyer 2018). These observations suggest that clarity and guidance are required at an antibiotic stewardship level.

The inappropriate prescription or the excessive use of antibiotics in dental practice could occur when antibiotics are prescribed for conditions that are not indicated for antibiotic prophylaxis such as dry socket, irreversible pulpitis, acute periapical infection and pulpitis, non-clinical factors such as patient expectations, convenience or for viral infections such as herpes simplex virus-1 infections (Agnihotry et al. 2016; Mthethwa & Matjila 2018; Sancho-Puchades et al. 2009). Alarmingly, the study findings suggested that antibiotics were prescribed for dental conditions that did not require such cover. Some participants in this study indicated that they would prescribe antibiotics for pain related to reversible and irreversible pulpitis, dental fillings, pericoronitis, periodontitis, impacted teeth, trismus and aphthous ulcers. These findings are consistent with Perić et al. (2015:111) who reported that antibiotics were prescribed as a precaution because of ‘uncertainty concerning the diagnosis, patient’s expectations, unavailability of dental services and in short-term cases where there is insufficient time for doing any treatment’. It is also noteworthy that a significant number of study participants (especially medical practitioners) were unsure of when to prescribe antibiotics for dental use. At the same time, shortages in the availability of oral healthcare workers and inadequate infrastructure for facility-based care in the public sector (Singh et al. 2010) could be the possible reasons for the reported ‘overprescription’ of antibiotics in this study. This could explain why study participants opted to prescribe antibiotics for dental-related pain instead of offering or referring for treatment services.

Interestingly, a pattern of antibiotic prescription also emerged in this study based on the clinical site where the respondent was located. These differences in prescription patterns for the same dental condition were surprising (Ramnarain 2021). More research is thus required to further understand these differences across clinical settings. One of the possible reasons for the reported difference in prescription patterns in this study could be professional territorialism where practitioners at one institution tend to follow a particular trend in prescribing antibiotics.

Gutiérrez et al. (2006), therefore, highlight the need for professional agreement and consensus building with regard to the conditions for antibiotic prescriptions. Such efforts are also needed in a South African context to facilitate practitioner consensus building to ensure consistency in antibiotic prescription for dental use (Ramnarain 2021). This highlights the need for more practitioner support through the use of developed guidelines on antibiotic prescriptions and collaboration at a multidisciplinary team level (Lalloo et al. 2017b; Perumal-Pillay & Suleman 2017).

It is concerning to note that while 81.8% of respondents reported the existence of a hospital-based antibiotic stewardship programme, yet less than half of the study sample (47.7%) were aware of the activities related to this initiative. This suggests an urgent need to strengthen antibiotic stewardship at an institutional level (Ramnarain 2021). Antimicrobial stewardship and infection and prevention control teams could provide opportunities to augment prescribing practices and streamline this process (South African National Department of Health 2015a,b; Ntšekhe et al. 2013).

Clearly, this study findings indicate serious shortcomings in the antibiotic prescription patterns for dental conditions and further highlight the obvious gaps to support medical and dental practitioners’ decision-making in this regard. It is, therefore, incumbent upon the National Department of Health to review its National Strategic Framework on Antimicrobial Resistance to include the oral health sector in the planning and implementation of guidelines for antibiotic prescription for dental conditions, as well as in other related activities associated with creating awareness on the need for reducing antimicrobial resistance (Ramnarain 2021). Oral health promotion and the awareness of antimicrobial resistance should be an important part of the healthcare service provision in the public and private healthcare facilities (Ramnarain 2021). National and international recommendations and facility-specific recommendations are required to combat antimicrobial resistance (Elias et al. 2017). The hospital pharmaco-therapeutic committees should be able to provide support and mentorship to the prescribing healthcare practitioner (Ntšekhe et al. 2013).

### Limitations of the study

This study provided valuable insight into the knowledge, practices and attitudes of healthcare practitioners regarding the trends in antibiotic prescription patterns for dental conditions. The generalisability of the study findings is limited to the study sites. There could have also been some over-reporting in respect of antibiotic prescription trends. More research is required to unpack the complexities associated with antibiotic prescription trends and patterns. The findings clearly emphasise an urgent need for clear evidence-based guidelines for healthcare practitioners that prescribe antibiotics for dental conditions (Ramnarain 2021).

## Conclusion

The results reveal that healthcare practitioners at the identified research sites reported inconsistent knowledge, attitudes and practices related to antibiotic prescription patterns for dental use. The study highlights the need for clear evidence-based guidelines for antibiotic prescription for dental conditions.
